# Obituary of Dr. S. Dunham

**Published:** 1865-02

**Authors:** 


### OBITUARY OF DR. S. DUNHAM.

At a called meeting of the St. Louis Dentists' Society, held at the office of the President, Dr. I. Forbes, Monday, the 20th February, 1865,

The Chair briefly announced that the meeting was called to adopt measures expressive of the loss sustained by the Society in the death of Dr. Sylvanus Dunham.

On motion, the President appointed Drs. H. E. Peebles, Aaron Blake and Henry Barron a committee to draft a suitable preamble and resolutions.

After consultation, the committee reported as follows.

Mr, PRESIDENT AND GENTLEMEN : Your committee beg leave to report that—

Whereas we have received the announcement of the death of Dr. Sylvanus Dunham, who expired on Saturday evening, the 18th instant ; and

Whereas, we had become strongly attached to the deceased by our long professional acquaintance, in the enjoyment of his valuable counsel and able advice; appreciating the modesty of his deportment and his many social virtues; but above all, the bright example of his Christian character; and

Whereas, it is proper, on occasions like the present, to give due expression to our feelings, under this afflictive dispensation of Divine Providence, by appropriate official actions; therefore be it

*Resolved*, That we have heard of the death of our friend, co-worker and brother, Dr. Sylvanus Dunham, with profound sorrow.

*Resolved*, That in the life and character of our deceased friend, we recognize a man well skilled in the nice and delicate manipulations of our profession; and possessing all these virtues which grace and enoble humanity; a friend, honest, honorable, sympathizing and steadfast. His heart and hand were ever open to the petitions of the poor.

*Resolved*, That we tender our heartfelt sympathies to the family and relatives of the deceased in their sad bereavement.

*Resolved*, That in token of our high appreciation of the character and warm esteem of the memory of the deceased member of our Society, we will attend his funeral in a body.

*Resolved*, That a page in our Record Book be set apart and appropriated to the special purpose of the record of his name, age and death.

*Resolved*, That these proceedings, signed by the President and Secretary, be communicated to the widow of the deceasad, and published in the principal daily papers of the city, and in the Dental Register of the West.

All of which is most respectfully submitted.

H. E. PEEBLES, D. D. S.,
AARON BLAKE, D. D. S.,
HENRY BARRON, D. D. S.,
Committee.

On motion of Dr. Spalding, the report was unanimously adopted.

After which, appropriate remarks were made by Drs. C. W. Spalding, H. E. Peebles, A. Blake, Isaac Comstock, and President Forbes, reviewing the life and labors, and eulogizing the character of the departed friend, and all bearing testimony to the sterling integrity and moral worth of the deceased; deeply regretting that one so useful and amiable had been taken from our select and faithful little Society, but humbly submitting to the all-wise judgment of that Divine Operator who "doeth all things well."

ISAIAH FORBES, D. D. S., President.

W. H. EAMES, D. D. S., Secretary.

